# Enhancers dysfunction in the 3D genome of cancer cells

**DOI:** 10.3389/fcell.2023.1303862

**Published:** 2023-11-06

**Authors:** Giulia Della Chiara, Carlos Jiménez, Mohit Virdi, Nicola Crosetto, Magda Bienko

**Affiliations:** ^1^ Human Technopole, Milan, Italy; ^2^ Department of Microbiology, Tumor and Cell Biology, Karolinska Institutet, Solna, Sweden; ^3^ Science for Life Laboratory, Solna, Sweden

**Keywords:** enhancers, enhancers hijacking, 3D genome organization, cancer, structural variants, epigenetics, 3D genomics, spatial genomics

## Abstract

Eukaryotic genomes are spatially organized inside the cell nucleus, forming a threedimensional (3D) architecture that allows for spatial separation of nuclear processes and for controlled expression of genes required for cell identity specification and tissue homeostasis. Hence, it is of no surprise that mis-regulation of genome architecture through rearrangements of the linear genome sequence or epigenetic perturbations are often linked to aberrant gene expression programs in tumor cells. Increasing research efforts have shed light into the causes and consequences of alterations of 3D genome organization. In this review, we summarize the current knowledge on how 3D genome architecture is dysregulated in cancer, with a focus on enhancer highjacking events and their contribution to tumorigenesis. Studying the functional effects of genome architecture perturbations on gene expression in cancer offers a unique opportunity for a deeper understanding of tumor biology and sets the basis for the discovery of novel therapeutic targets.

## Principles of 3D genome organization and functions

The spatial organization of eukaryotic genomes within the cell nucleus meets the necessity to fit very long DNA molecules into relatively small nuclei with an average diameter of 10 µm (Sun et al., 2000; Piovesan et al., 2019). This physical constraint has represented an increasing challenge for eukaryotes during evolution as genomes became larger and more complex (Fedoroff, 2012). As a result, cells have evolved mechanisms of genome folding and compaction through DNA interactions with both structural and regulatory proteins and with RNA (Cao et al., 2021). The discovery that the genome is non-randomly spatially organized dates back to the observation that, in interphase nuclei, chromosomes form discrete territories, which are non-randomly arranged with respect to the nuclear lamina and each other (Cremer and Cremer, 2010; Crosetto and Bienko, 2020). Following the advent of high-throughput chromosome conformation capture (Hi-C) (Lieberman-Aiden et al., 2009) and of high-throughput fluorescence *in situ* hybridization (FISH) techniques that allow for direct visualization of genomic regions as short as few kilobases (kb) across thousands of nuclei (Bouwman et al., 2022), as well as super resolution live-cell imaging (SRLCI) approaches that measure dynamics of chromatin contacts in space and time (Gabriele et al., 2022; Mach et al., 2022), our understanding of the principles of three dimensional (3D) genome folding has dramatically expanded. These methods allow us to study 3D genome organization across structural domains at different levels within the nucleus. At a coarse-grain level of organization, the genome is folded into so-called A/B compartments, which segregate euchromatin and actively transcribed elements (A compartments) away from heterochromatin and silenced regions (B compartments) (Lieberman-Aiden et al., 2009). Embedded in these compartments, we find megabase-sized topologically associating domains (TADs) (Dixon et al., 2012; Nora et al., 2012), representing DNA regions that preferentially interact with each other over the rest of the genome, whose boundaries are well labelled by the binding of CCCTC-binding factor (CTCF) or other insulator proteins. At a lower level of 3D genome architecture, relatively short-range chromatin loops bridging distal genomic regions, including enhancers and promoters, occupy TADs and sub-TADs domains, contributing to gene expression modulation (Popay and Dixon, 2022).

Regulation of gene transcription is accomplished through the integration of events at regulatory elements, that are proximal (promoters) and distal (enhancers) to gene transcription start sites (TSSs). More than 40 years after their discovery, enhancers are considered to play a central role in the spatio-temporal control of gene transcription, underlying human development and homeostasis (Banerji et al., 1981; Gasperini et al., 2020). Enhancers are short non-coding stretches of nucleosomes free DNA sequences that act as positive regulators of transcription via their ability to bind key proteins—transcription factors (TFs) and architectural proteins (i.e., CTCF, cohesins, mediators) — and complexes that control the expression of a target gene, in a location, distance- and orientation-independent manner (Banerji et al., 1981). Enhancers’ function is affected by chromatin context (i.e., histone-specific modifications of surrounding nucleosomes–H3K4me1 and H3K27Ac) (Della Chiara et al., 2021) and cell-specific TFs or stimuli in the environment in which they reside (Heinz et al., 2015). Indeed, the recruitment of specific TFs and chromatin mobility contributes to loops formation between enhancers and their target promoters, brought in close proximity in the 3D space. The mechanism and factors underlying enhancer-promoter (E-P) communication continue to be a subject of study, especially due to seemingly transient and dynamic nature of enhancers activation (Uyehara and Apostolou, 2023). Certainly, the advent of genetic engineering, single-cell methods, SRLCI approaches and 3D chromosome conformation capture-based technologies and their continuous improvement both on *wet* and *in silico* analysis, have pushed forward a deeper understanding of genome-wide mapping of E-P contacts, aiming at assigning one or multiple target genes to each enhancer, in time and space [for more details see (Brandão et al., 2021; Chen et al., 2023; Uyehara and Apostolou, 2023; Zhang et al., 2023)]. For example, intragenic enhancer at the *PVT1* locus gene interacts strongly with the *PVT1* promoter and weakly with the upstream *MYC* promoter gene. The inhibition of *PVT1* promoter increases *MYC* gene expression and *vice versa*. These transcriptional alterations are thought to be due to chromatin contact changes between these two promoters and the intragenic enhancer, highlighting how different configurations of 3D genome can affect E-P contact strength and specificity (Cho et al., 2018). More recently, two live-imaging studies focusing on the *sox2* and *eve* loci, in mouse embryonic stem cells (Alexander et al., 2019) and *drosophila* embryos (Chen et al., 2018), respectively, reached opposing conclusions on the correlation between the E-P proximity and gene transcription. These results highlight our incomplete understanding of the E-P communication and its relationship to gene expression. Needles to say, perturbations to 3D chromatin organization could be responsible for enhancer dysfunctions, resulting in the activation of alternative gene programs. These mechanisms are central to sustain many pathological conditions, including tumors.

## Genome architecture dysregulation in cancer

**FIGURE 1:**
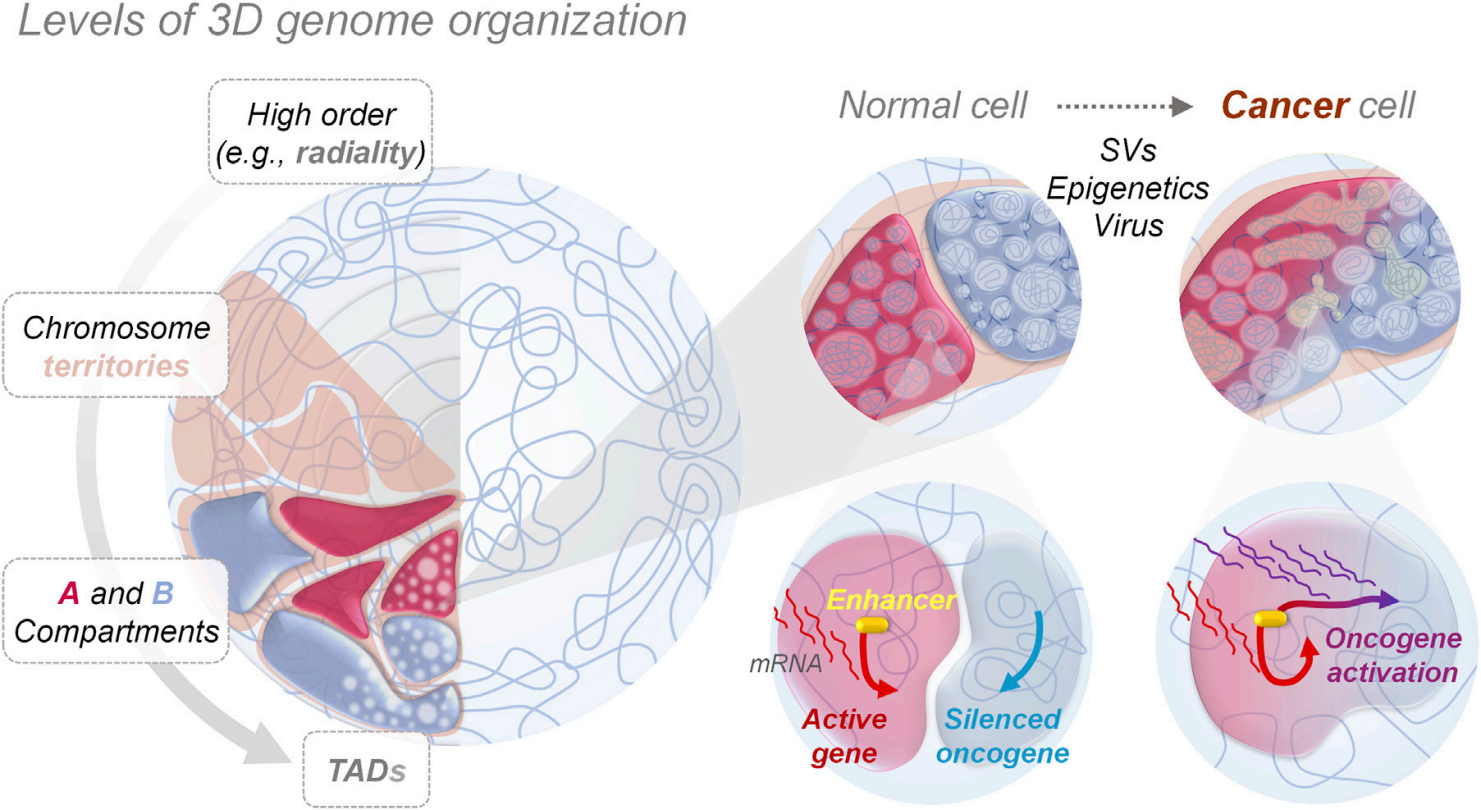
3D genome organization and aberrant genes activation. Nucleus in the left panel shows how genomes are orderly folded forming compartments at different levels, from big chromosome territories to smaller topologically associating domains (TAD), allowing the physical separation and performance of nuclear functions, such as gene expression programming. Zooms in the right panel show how, in cancer cells, genome mis-compartmentalization occurs at multiple levels, either for genetic (e.g., structural variants—SVs), epigenetic or environmental (e.g., viral infections, chemical agents) causes, playing a crucial role in aberrant gene expression, by altering the interaction between genes and regulatory elements like enhancers, thereby promoting and allowing tumorigenesis.

A variety of genetic and epigenetic mechanisms can disrupt 3D genome architecture and, in turn, cause aberrant gene expression in cancer cells, as summarized in Figure 1. Among genetic mechanisms, single-nucleotide variants (SNVs) or small insertions and deletions (indels) affecting the binding motifs or the structure of proteins implicated in establishing and/or maintaining the 3D genome structure—such as CTCF and cohesin—have the potential to rewire the 3D genome and gene expression, thus contributing to the cancer phenotype (Weisch et al., 2023). CTCF/cohesin-binding sites (CBS) are mutational hotspots in cancer (found primarily in gastrointestinal cancers), and somatic alterations to CBSs have been linked to altered expression of nearby gene (Katainen et al., 2015; Guo et al., 2018), likely due to altered CTCF-mediated looping. Moreover, topological stress at CBSs can create structural variants (SVs) (Canela et al., 2017), which in addition to SNVs, can have dramatic effects on 3D genome architecture and consequently gene expression. Among these two genomic rearrengements, SVs are the most common in various cancer types (Akdemir et al., 2020). They are a class of mutations encompassing losses, gains and rearrangements (inversions, duplications, or translocations) of DNA segments, ranging from few nucleotides to entire chromosomal arms (Currall et al., 2013), generated by improper repair of DNA double strand breaks (DSBs) (Aaltonen et al., 2020; Rheinbay et al., 2020). The analysis of 2,658 cancer samples originating from 38 tumor types from the International Cancer Genome Consortium (ICGC)/The Cancer Genome Atlas (TCGA) and Pan-Cancer Analysis of the Whole Genomes (PCAWG), has implicated SVs as tumor drivers in ∼62% of cases. Particularly, solid tumors seem to exhibit extremely high numbers of somatic SVs (253-310 SVs on average) (Li et al., 2020; Cosenza et al., 2022), revealing huge heterogeneity across patients, even within the same cancer type. Among all the SV types, deletions are the most common (26% of all SVs in the PCAWG dataset), and occur in the majority of tumors (Cosenza et al., 2022).

Cancer-associated SVs can disrupt the physiological 3D genome structure by causing A/B compartments switching (Dubois et al., 2022)or TAD reorganization (shuffling, fusion or neo-formation) (Lupiáñez et al., 2015; San Martin et al., 2021; Xu Z. et al., 2022), resulting in the ectopic activation of oncogenes or inhibition of tumor-suppressing genes (Liu et al., 2023). That is the case for several primary and metastatic prostate cancer cell lines, where SVs-mediated DNA loci transition from the inactive to active compartment and vice versa can cause the fusion of TMPRSS2-ERG *genes* - *a marker used for prostate cancer malignancy stratification -* (San Martin et al., 2021), and the activation of numerous genes linked to carcinogenesis (i.e., *WNT5*, *TMPRS*, and *CDK44*) (San Martin et al., 2021) (Table 1). Another example of SVs-mediated A/B compartments switching is observed in multiple myeloma, where translocation of the histone lysine methyltransferase gene *NSD2* from chromosome 4 to 14 highly increases its expression, leading to pervasive methylation of H3K36, with close TADs structure alteration and oncogenic genes pathways activation (Lhoumaud et al., 2019). One of the major consequences of SVs-mediated changes to 3D chromatin folding stems from repositioning (spatially or along the linear genome) of non-coding DNA regulatory elements (i.e., enhancers), affecting their interactions with target genes. This is because the specificity exhibited by enhancers is non-autonomous: if moved to a new locus they will generally activate any gene in their vicinity. This event called ‘enhancer hijacking’ has been increasingly identified as a driver of oncogenic behavior of structural variants (Ibrahim and Mundlos, 2020). Thus, determining how distant genomic regulatory elements, such as enhancers and promoters, communicate, is essential to any detailed understanding of transcriptional control in healthy and neoplastic development processes.

**TABLE 1:** Genomic rearrangements (SVs) induced enhancers hijacking events and their effect on gene expression alteration in different tumor types.

SVs	Malignancy		References
Compartments switching	Prostate Cancer	• TMPRSS2-ERG gene fusion	San Martin et al. (2021)
		• Upregulation of *WNT5a* and *CDK44*	
TAD disruption (Translocation)	Multiple Myeloma	• Overexpression of *NSD2* (Histone methyltransferase) drives compartment switching	Lhoumaud et al. (2019)
TAD fusion (Deletion)	T-ALL	• Upregulation of *MYC* transcription	Kloetgen et al. (2020)
TADs fusion/reshuffle	Colorectal Cancer	• Activation of *TOP2B* and *CHEK1* genes	Kim et al. (2023)
Neo-Chromatin Loop	T-ALL	• Altered gene expression of oncogenic transcription factors *HOXA*, *TLX3*, and *TAL2*	Yang et al. (2021)
	Pancreatic Cancer	• Upregulation of LIPC	(Roe et al., 2017; Ren et al., 2021)
	Acute Myeloid Lymphoma	• Upregulation of oncogenes *MYCN, WT1*, and *RUNX1*	Xu et al. (2022a)
Insertion, deletion	Medulloblastoma	• Proto-oncogenes *GFI* and *GFIB* activation	(Northcott et al., 2014; Northcott et al., 2017)
Insertion/Translocation	Acute Myeloid Lymphoma	• Overexpression of proto-oncogene *EVI1*	Gröschel et al. (2014)
Translocation	Angiocentric Gliomas	• Partial loss of *QKI* (tumor suppressor gene) expression due to *MYB-QKI* gene fusion	Bandopadhayay et al. (2016)

## DNA regulatory elements dysfunction in cancer: enhancer hijacking

In cancer, SVs such as chromosomal translocations as well as inversions and duplications, can lead to the formation of *de novo* E-P contacts, leading in turn to the activation of proto-oncogenes in a process called enhancer hijacking. In this process, there are two possible mechanisms: the sequence of an active enhancer is transferred closer to a different promoter (along the linear genome) reinforcing or activating its transcription. Alternatively, SVs can trigger a fusion between contiguous TADs, leading to the emergence of long-range contacts between a promoter of a tumor-driving gene from one TAD, and an enhancer from the second TAD that normally serves other gene(s) (Bienko, 2022; Sidiropoulos et al., 2022). Enhancers hijacking has been described in several tumor types such as medulloblastoma, pancreatic and thyroid cancers, multiple myeloma, mantle cell lymphoma, and several leukemias, causing dysregulation/activation of several proto-oncogenes (Gröschel et al., 2014; Bandopadhayay et al., 2016; Northcott et al., 2017; Ren et al., 2021; Rico et al., 2022) (Table 1). For example, in T-cell acute lymphoblastic leukemia (T-ALL) deletion of a TAD boundary fuses the neighboring TADs resulting in the abnormal interaction of the *MYC* gene promoter with the *BDME* gene enhancer (Kloetgen et al., 2020), inducing *MYC* transcription upregulation. Moreover, formation of new chromatin loops leads to novel E-P contacts that alter expression of oncogenic transcription factors *HOXA*, *TLX3*, and *TAL2* (Yang et al., 2021), contributing to T-ALL pathogenesis. Similarly, *de novo* chromatin loops formation creating unwanted promoter-enhancer interactions, causes upregulation of *LIPC* in pancreatic cancer metastasis (Roe et al., 2017; Ren et al., 2021) or upregulation of oncogenes such as *MYCN, WT1*, and *RUNX1* in acute myeloid lymphoma (Xu J. et al., 2022). In medulloblastoma brain tumors, enhancer hijacking, caused by SVs, is an efficient mechanism driving the activation of proto-oncogenes *GFI1* and *GFI1B*, moving their coding-sequences close to active enhancers (Northcott et al., 2014; Northcott et al., 2017). Recently, also in colorectal cancer (CRC) patients, SVs-mediated 3D genome dysregulation was found to cause super enhancers (SE) elements hijacking, leading to the hyper-activation of *TOP2B* and *CHEK1* genes. These genes are linked to genome instability and DNA repair mechanisms and their high expression is thought to sustain tumor cells survival and proliferation, making these SE-hijacking events tumor promoting, representing new potential therapeutic targets (Kim et al., 2023). Also in gastric cancers, an integrated paired-end NanoChIP-seq (PeNChIP-seq) combined with whole genome sequencing (WGS) approach revealed new tumor-associated regulatory SVs-mediated enhancers hijacking, in regions associated with both simple and complex genomic rearrangements. These genetic and epigenetic alterations were found to activate *CCNE2* and *IGF2* oncogenes in 8% and 4% cancer patients respectively, further highlighting how enhancers hijacking could help in the identification of novel actionable tumor targets (Ooi et al., 2020).

Interestingly, new several *in silico* tools have recently emerged to identify enhancer hijacking in cancer genomes. For example, the NeoLoopFinder (Wang et al., 2021) is a computational framework aimed at identifying hijacked enhancers by integrating H3K27ac ChIPseq data, DNase-seq for chromatin accessibility and RNA-seq with Hi-C matrix, SVs input list and WGS derived from several cancer cell lines. The resulting hijacked enhancers are labelled by H3K27ac peaks or DNase accessible regions and are found in anchors of the expected neo-loops connected to gene promoters. This method predicted enhancer hijacking events in 11 cancer cell lines (for details check Wang et al. (Wang et al., 2021)) and showed how genes connected to these adoptive enhancers were expressed at higher levels compared to the same genes analysed in control cell lines, further reinforcing the relevance of enhancer hijacking in cancer. Another tool for enhancer hijacking prediction is Activity By Contact or ABC (Xu Z. et al., 2022), which integrates chromatin contacts measured by Hi-C and enhancer activity detected by ChIP-seq to predict target gene expression, after enhancer hijacking events induced by SVs. Application of ABC to patient-derived tumor samples and various cancer cell lines identified multiple TAD fusion events causing highjacking of enhancers or super-enhancers, inducing the activation of *MYC*, *TERT* and *CCND1* oncogenes (Xu Z. et al., 2022). Future studies will be needed to determine whether tools such as NeoLoopFinder and ABC can be used in a clinical setting to identify targetable tumor drivers activated as a result of enhancer highjacking.

## Conclusions and future perspectives

The three-dimentional architecture of the genome is vital for gene expression regulation and its disruption by genetic or epigenetic alterations is emerging as an important contributor to the pathogenesis of cancer. Several mechanisms by which the 3D genome gets perturbed in cancer have been described, including mutations in proteins shaping genome architecture or in their binding motifs, rewiring of A/B compartments and TADs and enhancer hijacking. However, several major questions in this arena remain unanswered: are genomic rearrangements and consequent 3D genome architecture perturbation the only cause for enhancers hijacking? Can enhancer hijacking be caused by non-genetic mechanisms such as reactivation of transposable elements within specific genomic regions? Can specific nucleotide sequence features (i.e., Alu, GC content, etc.) affect enhancer hijacking? Are there any shared enhancers hijacking events among different tumor types? And, most importantly, can we target dysfunctional enhancers or aberrant TADs/loops for therapeutic purposes?

Answering these questions will require charting the 3D genome directly in patient-derived tumor samples and, integrating 3D genome measurements to WGS data and new *in silico* tools in clinical trials, will be useful to assess the diagnostic and predictive value of SVs and associated 3D genome changes. Ultimately, developing cost-effective and scalable approaches for screening the effect of drugs or genetic perturbations on 3D genome architecture will be needed to develop the first generation of 3D genome targeted therapies.
